# Has mental health changed in children and adolescents registered with a dedicated support service responding to the Manchester Arena attack: 3-year follow-up

**DOI:** 10.1192/bjp.2025.10310

**Published:** 2026-03

**Authors:** Louise Hussey, Matthew Gittins, Anna Hedges, Scott Dobbie, Alan Barrett, Paul French, Prathiba Chitsabesan

**Affiliations:** Division of Psychology & Mental Health, The University of Manchester, Manchester Academic Health Science Centre (MAHSC), Manchester, UK; National Institute for Health and Care Research (NIHR) Applied Research Collaboration – Greater Manchester (ARC–GM), The University of Manchester, Manchester, UK; National Institute for Health and Care Research (NIHR) Manchester Biomedical Research Centre, The University of Manchester, Manchester, UK; Centre for Biostatistics, Division of Populational Health, Health Services Research, and Primary Care, The University of Manchester, Manchester, UK; Greater Manchester Mental Health NHS Foundation Trust, Manchester, UK; School of Health and Society, University of Salford, Salford, UK; Research and Innovation Department, Pennine Care NHS Foundation Trust, Ashton-under-Lyne, UK; Division of Psychology and Language Sciences, University College London, London, UK; Pennine Care NHS Foundation Trust, Ashton-under-Lyne, UK

**Keywords:** Children and young persons, PTSD, depression, anxiety, post-trauma incidence response

## Abstract

**Background:**

The Resilience Hub was established to coordinate mental health and psychosocial support for anyone affected by the 2017 Manchester Arena terrorist attack.

**Aims:**

To use the Hub’s mental health screening data to examine the variation in symptoms reported by children and young persons (CYP) and their parent/guardian and explore any association with time delay in post-event registration or parental distress.

**Method:**

CYP engaging with Hub services were separated into eight ‘admission’ groups depending on when they registered post-incident. CYP were screened for trauma, depression, and generalised and separation anxiety. Parents/guardians also completed screening measures for their own and their child’s anxiety. Baseline and follow-up scores were compared between admission groups. Parental and CYP assessments of the CYP’s anxiety score was compared with the measure of parental distress.

**Results:**

Almost half of CYP registered in the first 3 months of service launch, with numbers of new registrations falling during each subsequent screening cycle. Generally, there was an increase in baseline screening scores as Hub registration time increased. The Children’s Impact of Event scale score decreased by 0.11 (95% CI: −0.17, −0.05) per month, but the score for depression increased by 0.06 (95% CI: 0.03, 0.10). Longitudinal patterns in anxiety and separation were difficult to discern. Screening scores of CYP registering later reduced at a faster rate than those of the first registrants. Higher levels of parental mental distress were correlated with increased anxiety scores assigned to the CYP in relation to the anxiety score reported by the CYP themselves.

**Conclusion:**

CYP who registered earlier were less symptomatic, although those registering later did show increased improvement in their symptoms, indicating that the Hub was beneficial. Parental well-being was associated with child mental distress, indicating that shared family trauma should be considered when planning care.

Adolescents experiencing trauma have been shown to be at increased risk of developing post-traumatic stress disorder (PTSD) and major depressive disorder.^1^ As a cause of trauma, terrorism can be distinguished from natural disasters in its capacity to generate a greater sense of fear, unpredictability and loss of sense of safety. It is, therefore, often associated with increased risk of developing mental health disorders^2^ and increased use of mental health resources.^3^ Within the first 6 months of the 11 September World Trade Center attack, 7% of a sample of New York school students had accessed community mental health services in relation to the attack, and a further 11% accessed resources within their school.^4^ Research into the impact of terrorism-specific trauma on children and adolescents is limited compared with such research in adults. However, systematic reviews have estimated a prevalence of PTSD of 47% among children exposed to war, as well as elevated levels of depression and anxiety disorders, at 43% and 27%, respectively.^5^ A study examining the trajectory of distress and recovery in adults following the 2017 Manchester Arena bombing further demonstrated associations between disruptions to close relationships, slower recovery and greater post-event distress.^6^ These findings are comparable with those of a study of PTSD trajectories in an adult population following the 2001 World Trade Center incident. This showed increased risks of PTSD in those with greater exposure as well as those with subsequent unmet mental health needs and low social integration.^7^

## The Manchester Arena bombing and the Greater Manchester Resilience Hub

On 22 May 2017, a suicide bomber detonated an improvised explosive device at the exit of the Manchester Arena as concert attendees were leaving, killing himself and 22 members of the public and injuring many more.^8^ The Greater Manchester Resilience Hub (the ‘Hub’) was established via a collaboration among four National Health Service mental health trusts and ‘hosted’ by Pennine Care NHS Foundation Trust within a few weeks of the attack to support delivery of a psychosocial and mental healthcare pathway.^9^ The Hub used a proactive programme of outreach and screening, using validated self-report measures. Those scoring within a clinical range were provided with evidence-based treatments suitable to their needs. In addition, all children and young persons (CYP) and their families were provided with telephone support, information, self-help resources, and access to a range of direct and indirect psychosocial support, including peer-support opportunities, psychosocial advocacy with education establishments and help with secondary stressors.^8,10,11^ Screening tools were used to identify symptoms of PTSD, generalised anxiety and depression. Individuals were contacted by a Hub clinician for further follow-up.^8,11^ The stepped-care approach (universal, targeted and specialist support) included psychoeducation on trauma responses, signposting to other self-help information and/or services, and referral to other treatment providers. The Hub provided extensive direct therapeutic support delivered through one-to-one interventions and a range of workshops and group events. The majority (80%) of those seeking support were from outside Greater Manchester; therefore, in addition to clinicians travelling to those affected, help was provided to assist individuals in accessing local services.^8,11^

## Aims

We aimed to explore the variation and changes in mental health screening data submitted by CYP and their parent, guardian or relative registering with the Hub over the first 3 years of the Hub service launch. We also aimed to examine how Hub interventions were associated with mental health outcomes of CYP registrants by conducting an assessment of their contact with the service. We aimed specifically to answer the following questions.

- Were patient-reported mental health screening outcomes associated with the time taken to register with the Hub, and did the rate of change in these outcome measurements differ with the ‘time taken to join’?
- How did the self-reported screening scores of the CYP registrants compare with those completed by a parent, guardian or relative (in relation to that CYP) also registered with the Hub? Did the mental well-being of the parent, guardian or relative influence how they perceived the psychological health of their CYP?
- How did the length of contact time with the service relate to mental health outcomes?

## Method

### Study population and mental health screening measures

Upon joining the Hub, registrants completed diagnostic mental health screening questionnaires to provide baseline mental health scores, as well as a set of follow-up mental health scores at regular intervals after the event: every 3 months in the first year and then every 6 months for 3 years thereafter. Many of the acute, complex cases identified during the immediate aftermath of the arena event were not captured in the screening programme. Many of the bereaved and physically injured were already in receipt of bespoke packages of care with the Hub, having been referred by police and acute hospitals before the roll out of the screening, which was intended to identify the larger number of psychologically injured persons. CYP registrants (aged 16 years and under) completed three measures: two as part of the Revised Children’s Anxiety and Depression Scale^12^ and one for trauma (the Children’s Impact of Event (IES) scale).^13^ In addition, parents, guardians or relatives of the CYP completed two measures of the parental version of the Revised Children’s Anxiety and Depression Scale, one that provided an assessment of the CYP’s anxiety and another that gave a measure of separation anxiety.^14^ Here, the five measures are abbreviated as follows: CYP-GAD (CYP measure of generalised anxiety disorder), DEP (depression), IES (trauma), PG-GAD (parental measure of generalised anxiety disorder) and SEP (separation anxiety). This study replicated some of the methodology used in a previous study conducted within the first year post-event.^8^

### Baseline admission groups

The mental health screening measures were completed on a voluntary basis; therefore, the number of measures recorded for each registrant was dependent on the level of engagement and length of time engaged with the service. All Hub registrants with at least one mental health screening measure were included in our study. To assess the impact of the time taken to join the Hub, at baseline admission, registrants were grouped into eight admission groups according to the time frame in which they joined.^8^ The baseline admission groups, which started with registrations occurring 3 months post-event, were defined as follows: admission group 1: 3–6 months, from 9 September 2017 to– 20 November 2017; admission group 2: 6–9 months, from 21 November 2017 to 15 February 2018; admission group 3: 9–12 months, from 16 February 2018 to 10 May 2018; admission group 4: 12–18 months, from 11 May 2018 to 15 October 2018; admission group 5: 18–24 months, from 16 October 2018 to 10 April 2019; admission group 6: 24–30 months from 11 April 2019 to 8 October 2019; admission group 7: 30–36 months from 9 October 2019 to 9 June 2020; admission group 8: 36+ months (36–42 month period) from 10 June 2020 to 31 August 2020.

All mental health screening scores were downloaded from the Hub’s patient clinical information system using a unique anonymised client ID. Although this case management system contained some demographic and event-related information, this information was not recorded for all individuals; therefore, CYP mental health practitioners reviewed case-note recording information. This included age at registration, gender, presence at the Arena (yes or no), a record of a previous mental health condition (yes or no) and a measure of the individual’s exposure to physical injury (no, yes – some minor injury, or yes – significant major injury).

### Analysis

We compared mental health scores during follow-up across the eight baseline admission groups to explore whether mental health score was associated with time to registration post-event. Using a STATA v.16 mixed model, we fitted a multi-level mixed-effects linear regression model with a random intercept for each individual mental health score. The random effects intercept accounted for within-individual versus between-individual variation in health scores by clustering responses by participant ID. For each mental health score (i.e. the dependent variable) a set of predefined fixed effects covariates were included. These were admission group (1–8), follow-up time in months to each follow-up mental health score, and baseline mental health score. These were subsequently further adjusted for pre-identified confounders, age at registration, gender, presence at the Arena and previous recorded mental ill health. Finally, an interaction term between admission group and follow-up time was used to investigate whether the change in mental health score during follow-up differed among admission groups.

### Parent/guardian and CYP anxiety screening scores

To assess any relationship between the CYP anxiety scores reported by the CYP and those reported by the parent, guardian or relative at baseline, we subtracted the baseline PG-GAD score from the baseline CYP-GAD score, creating a CYP–PG-GAD variable. A negative value of this score indicated that the parent, guardian or relative scored the CYP’s level of anxiety higher than the CYP scored themselves. The relationship between the parent, guardian or relative’s own screening scores and the difference in CYP–PG-GAD score was also assessed to ascertain whether mental distress of the parent, guardian or relative showed any correlation with the anxiety score recorded for their CYP.

### Contact time with the Hub

All appointments and forms of contact with the Hub were recorded, including the number of minutes of ‘contact time’ experienced by the participant. Using these data, we described the type of appointment and explored any association with mental health outcomes. The number of cumulative minutes of ‘contact time’ by each follow-up assessment were included as an explanatory variable in the mixed-effects model described previously.

### Ethics statement

This study was part of a service evaluation strategy using routinely collected data. As such, ethical approval and patient consent were not sought.

## Results

Overall, 710 CYP registered with the Hub between May 2017 and August 2020 and subsequently completed at least one CYP screening measure. Almost half (329; 46%) registered within the first 3 months after the Arena attack.

Table 1 describes the eight admission groups by demographic characteristics. Participants were predominantly female (612 of 710; 86%) and most frequently in the 13–14 year age group (246; 35%). The mean age was 13.31 years (s.d. 1.91). Nine per cent (61 individuals) of the CYP population had case-note information pertaining to a previous mental health condition, and the majority (633; 89%) were known to have been present at the Arena during the incident. Across the eight admission groups, as time taken to join the Hub increased, there was a general increase in the proportions of individuals that were male and had evidence of a pre-existing mental health condition. Conversely, there was a general decrease in the proportion of people present at the Arena on the evening of the attack.

**Table 1:** Demographic characteristics and covariates included in analysis by admission group of the CYP Hub registrants

	Admission group (time to register with the Hub after the Arena event)								
	Group 1	Group 2	Group3	Group 4	Group 4	Group 5	Group 7	Group 8	
	3–6 months	6–9 months	9–12 months	12–18 months	18–24 months	24–30 months	30–36 months	36–42 months	Total
Registrants	*n* (%)	*n* (%)	*n* (%)	*n* (%)	*n* (%)	*n* (%)	*n* (%)	*n* (%)	*n* (%)
All Hub registrants	329 (46.3)	108 (15.2)	79 (11.1)	65 (9.2)	49 (6.9)	35 (4.9)	23 (3.2)	22 (3.1)	710 (100.0)
Gender									
Female	306 (93.0)	100 (92.6)	71 (89.9)	62 (95.4)	34 (69.4)	27 (77.1)	11 (47.8)	1 (4.5)	612 (86.2)
Male	23 (7.0)	6 (5.6)	7 (8.9)	3 (4.6)	7 (14.3)	8 (22.9)	1 (4.3)	0 (0.0)	55 (7.7)
Other or not known	0 (0.0)	2 (1.9)	1 (1.3)	0 (0.0)	8 (16.3)	0 (0.0)	11 (47.8)	21 (95.5)	43 (6.1)
Total	329 (100.0)	108 (100.0)	79 (100.0)	65 (100.0)	49 (100.0)	35 (100.0)	23 (100.0)	22 (100.0)	710 (100.0)
Age group, years									
<10	16 (4.9)	8 (7.4)	2 (2.5)	3 (4.6)	3 (6.1)	2 (5.7)	0 (0.0)	1 (4.5)	35 (4.9)
10 to 12	73 (22.2)	29 (26.9)	24 (30.4)	16 (24.6)	13 (26.5)	8 (22.9)	2 (8.7)	0 (0.0)	165 (23.2)
13 to 14	121 (36.8)	47 (43.5)	23 (29.1)	23 (35.4)	18 (36.7)	11 (31.4)	3 (13.0)	0 (0.0)	246 (34.6)
15 to 16	119 (36.2)	24 (22.2)	30 (38.0)	23 (35.4)	8 (16.3)	14 (40.0)	7 (30.4)	0 (0.0)	225 (31.7)
Not known	0 (0.0)	0 (0.0)	0 (0.0)	0 (0.0)	7 (14.3)	0 (0.0)	11 (47.8)	21 (95.5)	39(5.5)
Total	329 (100.0)	108 (100.0)	79 (100.0)	65 (100.0)	49 (100.0)	35 (100.0)	23 (100.0)	22 (100.0)	710 (100.0)
Previous mental ill health									
Yes	20 (6.1)	13 (12.0)	5 (6.3)	8 (12.3)	7 (14.3)	8 (22.9)	0 (0.0)	0 (0.0)	61 (8.6)
No	305 (92.7)	89 (82.4)	72 (91.1)	56 (86.2)	34 (69.4)	25 (71.4)	11 (47.8)	1 (4.5)	593 (83.5)
Not known	4 (1.2)	6 (5.6)	2 (2.5)	1 (1.5)	8 (16.3)	2 (5.7)	12 (52.2)	21 (95.5)	56 (7.9)
Total	329 (100.0)	108 (100.0)	79 (100.0)	65 (100.0)	49 (100.0)	35 (100.0)	23 (100.0)	22 (100.0)	710 (100.0)
Present at the Arena									
Yes	325 (98.8)	98 (90.7)	70 (88.6)	59 (90.85)	41 (83.7)	29 (82.9)	10 (43.5)	1 (4.5)	633 (89.2)
No	2 (0.6)	0 (0.0)	6 (7.6)	1 (1.5)	4 (8.2)	3 (8.6)	1 (4.3)	0 (0.0)	17 (2.4)
Not known	2 (0.6)	10 (9.3)	3 (3.8)	5 (7.7)	4 (8.2)	3 (8.6)	12 (52.2)	21 (95.5)	60 (8.5)
Total	329 (100.0)	108 (100.0)	79 (100.0)	65 (100.0)	49 (100.0)	35 (100.0)	23 (100.0)	22 (100.0)	710 (100.0)
Exposure to trauma									
Yes – significant major trauma	4 (1.2)	5 (4.6)	2 (2.5)	2 (3.1)	1 (2.0)	0 (0.0)	0 (0.0)	0 (0.0)	14 (2.0)
Yes – some minor injury	13 (4.0)	8 (7.4)	3 (3.8)	5 (7.7)	3 (6.1)	0 (0.0)	0 (0.0)	0 (0.0)	32 (4.5)
No	305 (92.7)	86 (79.6)	59 (74.7)	47 (72.3)	37 (75.5)	10 (28.6)	0 (0.0)	0 (0.0)	544 (76.6)
Not known	7 (2.1)	9 (8.3)	15 (19.0)	11 (16.9)	8 (16.3)	25 (71.4)	23 (100.0)	22 (100.0)	120 (16.9)
Total	329 (100.0)	108 (100.0)	79 (100.0)	65 (100.0)	49 (100.0)	35 (100.0)	23 (100.0)	22 (100.0)	710 (100.0)

**Fig. 1:**
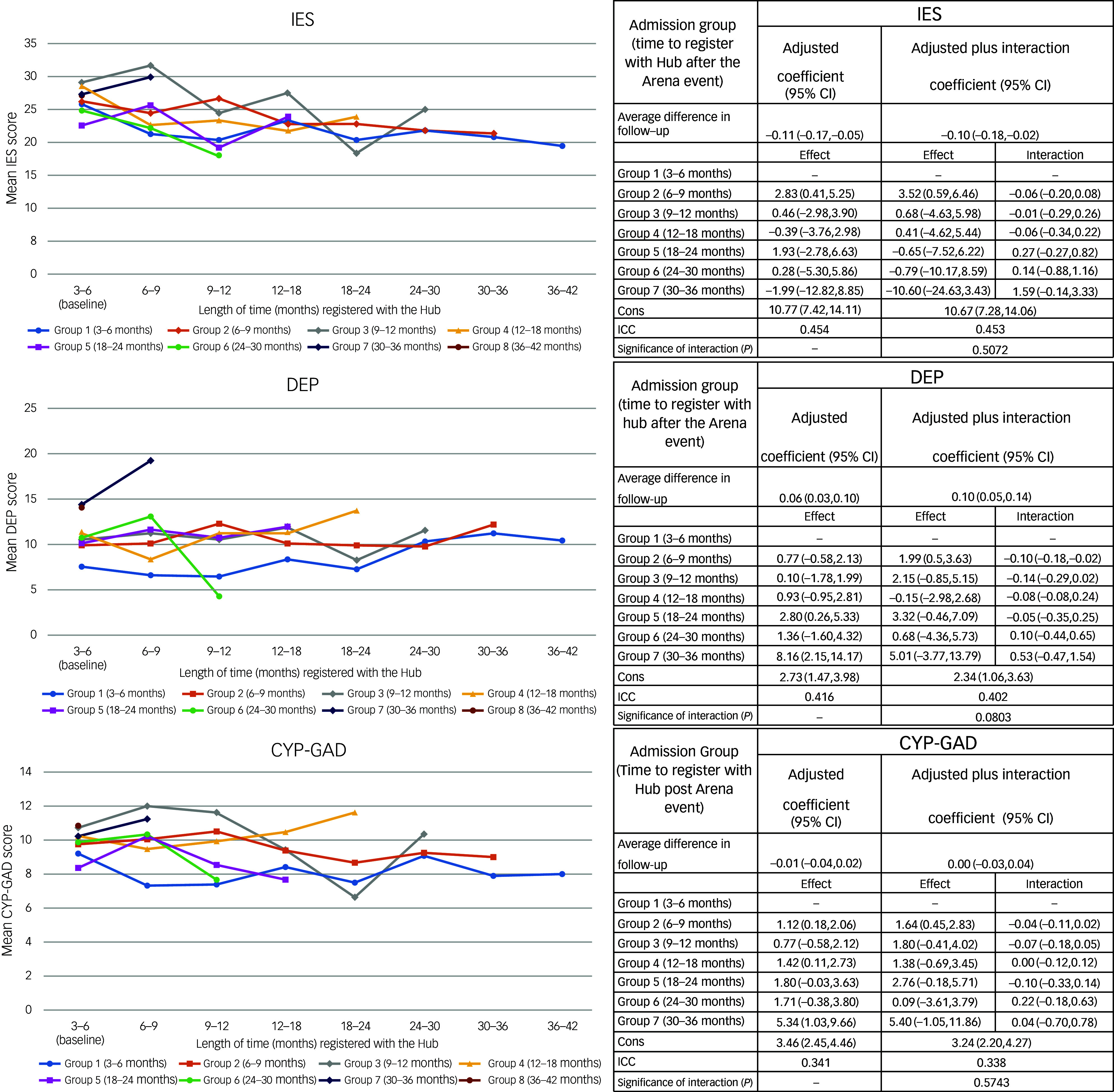
Plots showing mean change in score with length of time registered with the Hub and mixed regression models examining relationships of time between Arena event and joining Resilience Hub (admission group) with total Children’s Impact of Event scale (IES), Revised Children’s Anxiety and Depression Scale (DEP) and children and young persons generalised anxiety disorder scale (CYP-GAD) scores during follow-up. Cons, intercept term for the model indicating the predicted value of the outcome variable when all other predictor variables are set to zero. ICC, interclass correlation coefficient: the ratio of within subject variation to total variation, i.e. the amount of variation explained by differences in data subjects.

**Fig. 2:**
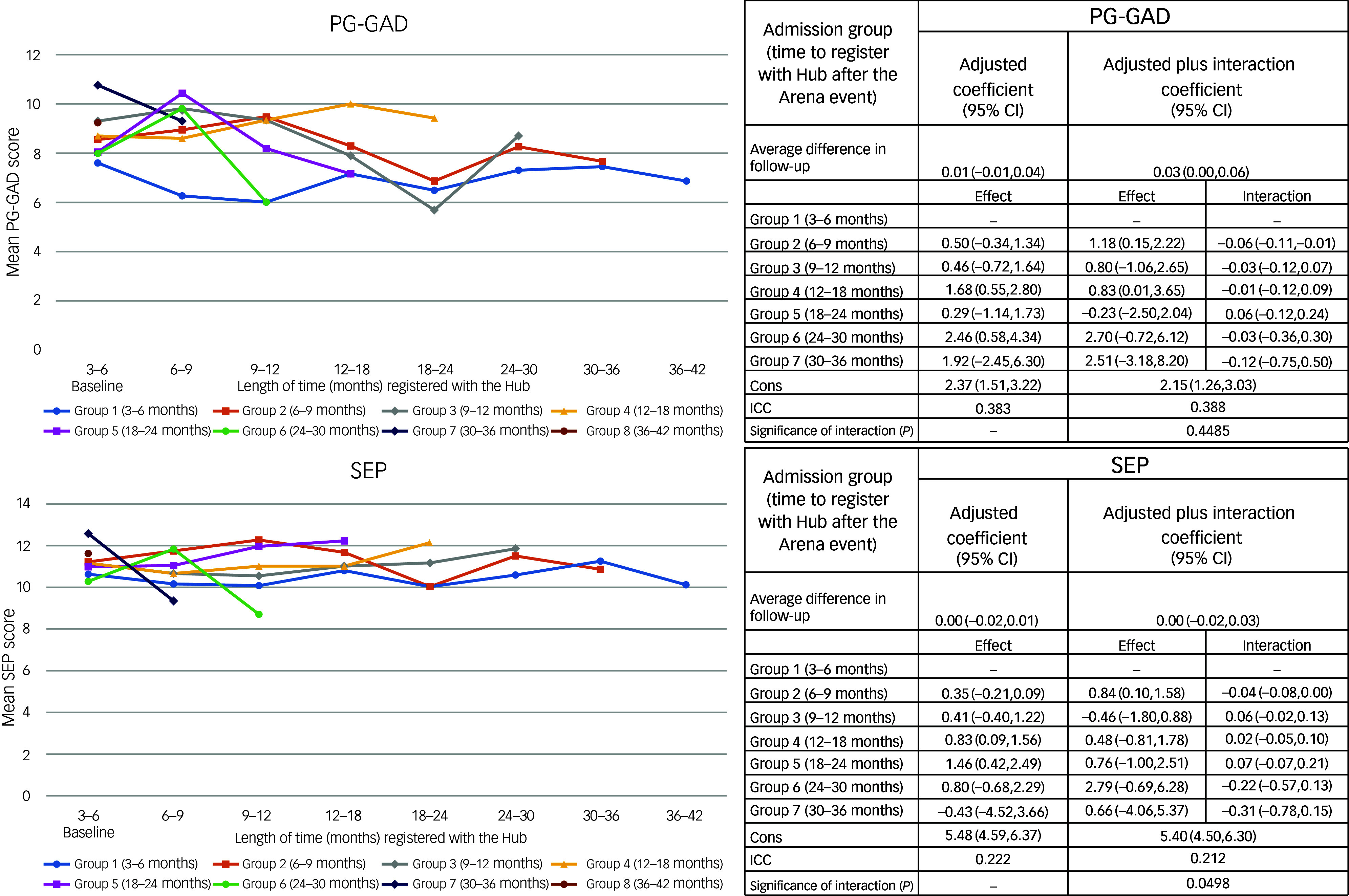
Plots showing mean change in score with length of time registered with the Hub and mixed regression models examining relationships of time between Arena event and joining Resilience Hub (admission group) with total parent-, guardian- or relative-reported generalised anxiety disorder scale (PG-GAD) and separation anxiety (SEP) scores during follow-up. Cons, intercept term for the model indicating the predicted value of the outcome variable when all other predictor variables are set to zero. ICC, interclass correlation coefficient: the ratio of within subject variation to total variation, i.e. the amount of variation explained by differences in data subjects.

The results of the multi-level mixed-effects regression model for follow-up mental health scores since the first measure recorded after joining the Hub (baseline), adjusted for covariates, are shown in Figs. 1 and 2. The results of the unadjusted models are presented in Supplementary Tables 2 and 3 available at https://doi.org/10.1192/bjp.2025.10310). As time (in months) taken post-event to register with the Hub increased, DEP, CYP-GAD, P-GAD and SEP scores (Figs. 1 and 2) generally increased, although confidence intervals were wide. The interaction effects between baseline groups and follow-up time were not statistically significant, indicating no evidence that mental health scores over follow-up were different within admission groups.

IES scores decreased on average by 0.11 (95% CI: −0.17, −0.05) per month of follow-up since the CYP registered with the Hub, regardless of admission group. Compared with those joining the Hub between 3 and 6 months post-event, the average follow-up score peaked in those joining the Hub between 6 and 12 months (2.83; 95% CI: 0.41, 5.25). During follow-up, DEP scores increased by 0.06 (95% CI: 0.03, 0.10) per month (Fig. 1). Compared with those joining between 3 and 6 months, the average follow-up DEP score was greater for all other admission groups (although the difference was generally not statistically significant). As illustrated in Figs. 1 and 2, there appeared to be no distinct change in longitudinal GAD follow-up scores for measures completed by either the CYP themselves or by their parent, guardian or relative. The baseline scores for all admission groups were generally higher than those of the 3−6 month group, illustrating a possible increase in general anxiety disorder symptoms among later registrants. The interaction model indicated that most admission groups showed a slightly stronger decrease per month of follow-up in GAD scores compared with the 3−6 month group, suggesting that GAD scores completed by both CYP and adults fell at a slightly increased rate. However, the interaction was not statistically significant. SEP scores also indicated no change over follow-up, and although the interaction was borderline statistically significant, the pattern was not clear across admission groups (Fig. 2).

### Comparison with parent/guardian screening in baseline GAD scores

**Fig. 3:**
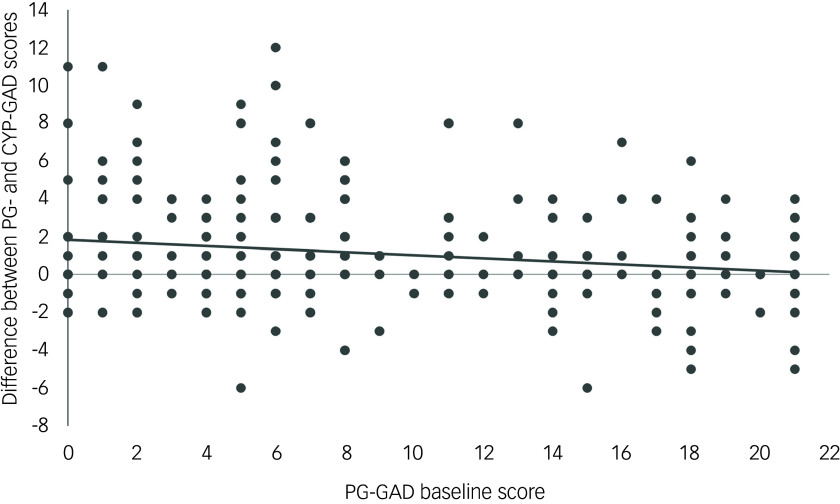
Differences between parent-, guardian- or relative-reported generalised anxiety disorder scale (PG-GAD) and children and young persons generalised anxiety disorder scale (CYP-GAD) baseline scores according to measures of parent, guardian or relative’s anxiety.

We identified 275 CYP registrants for whom we also had screening outcomes for anxiety reported by their parent, guardian or relative. The GAD-7 anxiety measure was used to assess the adult’s own anxiety, as described in a paper discussing mental health outcomes for adults registered with the Hub.^15^ The difference between the CYP-GAD and PG-GAD scores at baseline (CYP–PG-GAD) ranged from −6 to 12 (mean = 1.14; s.d. = 2.81). The proportion of parents, guardians or relatives with a positive CYP–PG-GAD score, indicating that they assigned an anxiety score to their child lower than the child scored themselves, was 48% (131 of 275). Twenty-nine per cent (80 of 275) of parents, guardians or relatives scored the CYP’s anxiety the same as the CYP themselves, 23% (64 of 275) had a negative score, meaning that the parent, guardian or relative rated the anxiety of their CYP higher than the child themselves. The correlation coefficient between the adult measure of anxiety (GAD-7) and the CYP–PG-GAD variable was −0.26 (Fig. 3), indicating that higher parent, guardian or relative anxiety was correlated with an increased anxiety score assigned to the CYP by the parent, guardian or relative.

### Contact time

A total of 6398 points of contact between CYP registrants and the Hub team were recorded; 73.8% were attended appointments (i.e. not cancelled or not attended) and therefore included in the analysis. The majority of these points of contact (91.7%) were for contacts categorised as assessment and/or triage (which also included elements of intervention, formulation and support). Supplementary Table 1 shows the frequency and mean number of minutes for each appointment type. Group therapy sessions and family support days were by far the longest (mean 268.4 min) forms of contact, followed by ‘treatment only’ appointments (21.1 min).

We calculated the mean contact time per person by dividing the total number of minutes spent in any contact with the Hub and the number of months actively registered (time between first and last date of contact) with the service. The 18–24 and 24–30 month admission groups had the highest mean contact times, with or without inclusion of group therapy sessions (41.1 and 35.4 min per month, respectively; 26.4 and 25.5 min without group therapy included). This did not, however, appear to be related to the mean number of appointments per person, which was lower than in other groups (7.1 appointments per person (18 months) and 7.7 (24 months)). Analysis of the number of cumulative minutes (for all appointment types) using mixed-effects linear regression modelling showed that increased contact time with the Hub was associated with a decrease in scores for all mental health screening measures. However, borderline significance was found only for DEP score (−0.090 (95% CI: −0.186, 0.002), *p* = 0.05). Results for the other four screening measurements were as follows: IES, −0.024 (95% CI: −0.144, 0.100), *p* = 0.74; CYP-GAD, −0.024 (95% CI: −0.096, 0.048), *p* = 0.48; PG-GAD, −0.036 (95% CI: −0.096, 0.024), *p* = 0.26; SEP −0.004 (95% CI: −0.048, 0.042), *p* = 0.88.

## Discussion

### Summary of main findings

Almost half of all CYP participants accessing the Hub registered in the first 3 months of the service launch (3–6 months after the Arena event), followed by an expected fall in the numbers newly registering in each admission group. Given the nature of the concert at the Arena on 22 May 2017 (Ariana Grande) the attendees (and subsequent Hub registrants) were predominantly female, and approximately two-thirds were aged between 13 and 16 years.^16^ Baseline mental health screening scores (DEP, CYP-GAD, PG-GAD and SEP) were generally greater as the admission time since the event increased, showing that people who registered earlier were less symptomatic. Over the 3 years of follow-up, there was a decrease in follow-up IES score, measuring the symptoms of trauma, but an increase in follow-up screening scores for symptoms of depression. The mean contact time per month was greatest for those registering with the Hub more than a year post-event (18–24 and 24–30 months admission groups). This is likely to reflect the fact that those who registered later were more symptomatic. It is also possible that this finding resulted from development of the services provided by the Hub. Our results also describe how parental well-being is related to the perceived well-being of their CYP. When there was a higher level of parent, guardian or relative mental distress, there was an increase in the anxiety score the parent, guardian or relative assigned to their CYP compared with the level of anxiety reported by the CYP themselves.

### Discussion of results in relation to the published literature

#### Time taken to seek support and engage with services

In keeping with previous findings examining Hub data,^8,15^ we found evidence of a general increase in baseline screening scores for depression, anxiety and trauma as time to register with the Hub increased. This suggests that CYP registering later were more symptomatic. One hypothesis for this finding is that owing to different health-seeking behaviours, those that are more proactive and engage with support services earlier have less severe symptoms. Individuals less willing to seek help are likely to do so as a result of symptom severity. A study examined the emotional reactions of parents of children who witnessed the 2011 Utøya shooting. Survivors who did not participate in initial interviews (at 4–5 months) were found to be more symptomatic when interviewed later (at 14–15 months).^17^ It is also possible that symptoms had not yet fully emerged, but untreated symptomatology worsened as time progressed. McFarlane and Papay also found that symptoms in children exposed to a bushfire at 2 months were less severe than those in the control group, and symptoms gradually increased over the next few months.^18^ This was further corroborated by our finding that people registering later with the Hub required more contact time with staff, suggesting that their symptoms were more severe or the case was more complex, and they therefore required more clinical intervention. Owing to the nature of the Arena event, there was an unusually high number of CYP involved for a terrorist incident. There is limited evidence available on the trajectory of disorder in CYP in major incidents. However, we know that in adults, the effects of trauma may be worse if left untreated. As such, those with greater symptom severity may register later but also experience worsening of symptoms the longer they take to seek help.^19^ In addition, symptoms may reduce over time for people engaging earlier owing to a therapeutic effect of registering with the Hub. Stancombe and Williams^6^ found (in adults) an indirect relationship between connecting with and receiving support from others who were also present at the Arena attack and mental well-being 3 years later, suggesting the importance of identifying with people who have shared experiences.

#### Trajectory of distress post-event

The post-disaster psychological response to trauma can take various forms. Although this will present as acute stress reactions in most cases,^20^ other chronic and pervasive serious mental health conditions can result from exposure to trauma.^21^ Overall, our results showed a monthly decrease in the measure of distress following trauma (IES score), suggesting a potential gradual improvement in mental well-being post-event. This is in line with previous research examining the trajectory of PTSD following the 2001 World Trade Center disaster. The authors of that study found associations with a number of factors, including low social integration and unmet mental health needs.^7^ The goals of the Hub included working to address these issues; therefore, we could expect to see potential improvements in IES scores over time. The increase in screening scores for depression was in keeping with the findings of a study examining the trajectory of depressive symptoms in adolescent survivors of the Wenchuan earthquake in China. Participants showed an increase in prevalence of depressive symptoms from 6 to 24 months and an increase in symptom prevalence at both anniversaries of the event.^22^

#### Engagement with Hub services

A systematic review examining natural PTSD recovery (i.e. without clinical intervention) 1 year post-trauma in CYP found that the prevalence reduced by approximately 50% in the first 6 months, with little evidence of further change thereafter.^23^ However, the authors noted that the majority of the studies included were of accidental injury and argued that this evidence base may not be applicable to ‘intentional trauma exposures’ such as terrorist attacks. Previous research on populations affected by disaster have demonstrated that within the first year, few people will spontaneously present for treatment, and for many there is a delay in seeking assistance and engagement with services, driven by the need for support for worsening symptomology with time.^24^ Our research also found that the mean contact time per month was greatest for those registering with the Hub more than a year post-event (18 month and 24 month admission groups). This is likely to be a reflection of the fact that those who entered later were more symptomatic and therefore driven to access help and, once in contact with the Hub, in need of greater support.

#### Association between CYP and parental mental well-being

Most Hub contact was time spent in provision of psychosocial support to the child in the presence of a caregiver, either at individual appointments or during group and/or family therapy sessions. In a smaller number of cases, CYP accessed group interventions from the Hub without caregivers present. Much of the one-to-one therapy was delivered locally to the CYP by existing commissioned services. Anecdotally, clinicians seeing children at the Hub sometimes reported that parents did not seek (or delayed seeking) support for their own mental health. This is consistent with previous studies showing that although parents of children being treated for mental distress experience mental disorders at a higher rate than the general population, they are unlikely to access mental health services themselves.^25,26^ Previous research has also found that parents of children exposed to terror attacks experienced major depressive and anxiety disorders at rates three times greater than those within the general population. In addition, rates of PTSD were reported as being five times greater.^27^ Within the Hub, parents often stated that they wished to prioritise their child’s mental health treatment before accessing support themselves. Parental mental health is a robust predictor of offspring mental distress, and screening of parental well-being in children’s mental health treatment can lead to parents accessing support for themselves. This has the potential to have a positive impact on their child’s treatment outcomes.^25,26,28^ We found that higher levels of parental mental distress could increase the anxiety score the parent assigned to their CYP compared with the level of anxiety reported by the CYP themselves. It is possible that the individual experience of mental distress affects decision-making and value estimation.^29^

### Strengths and limitations

This study used a unique data-set that has enabled analyses of longitudinal mental health screening data following a major traumatic incident. In addition to data for the CYP attending the Arena event, it includes screening data for friends and/or relatives, enabling further exploration of the impact of shared trauma upon the family unit. The use of multi-level modelling enabled clustering around patient case IDs, which allowed multiple screening cycle results to be associated with individuals, as participants are likely to have different thresholds in the way they report their symptoms. The data collected within the Hub was designed principally for clinical monitoring and not research purposes and therefore had some limitations. It was a convenience sample (Hub registrants) subject to demographic bias, which limited the external validity. In addition, demographic and event information was not consistently recorded, limiting examination of outcome predictors. We also lacked baseline pre-event screening data and comparative control groups. With the data available, we were unable to differentiate between the levels or types of treatment provided to Hub registrants. However, the analysis including contact time involved assessment of the amount of time individuals spent engaged with staff to receive treatment or assessment. In addition, we did not have data on external treatment as a result of referrals to other psychological services received or the impact of these on treatment outcomes. After the first 2 years post-incident, the number of people registering with the Hub declined. This resulted in small sample sizes for the 30 and 36 month admission groups and subsequent limits to data interpretation.

### Future implications

This study builds on the evidence base examining the role of timely access to mental health support for those affected by large-scale traumatic events and provides insight for future service provision aimed at improving outcomes for CYP. The results support the premise that being registered with services such as the Resilience Hub is associated with improvements in subjective distress over time. Our findings also suggest that treating the family unit as a whole is important in providing support for CYP who have experienced traumatic events. We acknowledge the limitations of this data-set, as primarily a clinical resource, and suggest it could be helpful in future to consider methods of data collection as soon as practicable during the creation of support services. There is more to be learned from the experiences of families who shared this trauma, and we recommend further research in this area. It would be beneficial to explore the impact of parental mental distress upon the CYP experiencing trauma and their trajectory of recovery.

## Supporting information

Hussey et al. supplementary material Hussey et al. supplementary material

## Data Availability

Owing to the sensitive nature of the data, the research data cannot be shared publicly.
