# Supplementary material for: Has mental health changed in children and adolescents registered with a dedicated support service responding to the Manchester Arena attack: 3-year follow-up
Source: Br J Psychiatry. 2025 Jul 17;228(3):244–51. doi: 10.1192/bjp.2025.10310 (PMC12912874; doi:10.1192/bjp.2025.10310)
Supplement: Hussey et al. supplementary material [file S0007125025103103sup001.pdf]

Supplementary Materials

Table S1. Contact time with the Hub by type of appointment and admission group

Admission Group	Mean number of months actively registered per person	Mean contact time per person (minutes)	Mean contact time per person w/o Group Therapy (minutes)	Mean number of appointments per person	Appointment Type (Number of appointments)				
					Assessment/ Triage	Treatment Only	Review Only	Group Therapy Session/Family Days	Total
(Number of Hub reguistrants in admission group)	N (SD)	N (SD)	N (SD)	N (SD)	N (%)	N (%)	N (%)	N (%)	N (%)
Mean (SD) number of minutes per appointment	_	_	_	_	19.2 (30.3)	21.1 (17.6)	15.6 (13.1)	268.6 (120.6)	28.7 (60.3)
Group 1 (3-6m)	16.7 (14.7)	26.7 (58.4)	17.8 (23.9)	6.0 (9.2)	1683 (92.7)	70 (3.9)	7 (0.4)	55 (3.0)	1816 (100.0)
Group 2 (6-9m)	17.4 (15.3)	28.4 (58.9)	18.4 (24.4)	9.3 (10.9)	953 (92.0)	39 (3.8)	0 (0)	44 (4.2)	1036 (100.0)
Group 3 (9-12m)	16.2 (14.9)	28.0 (58.8)	18.7 (35.0)	6.5 (5.9)	470 (92.5)	12 (2.4)	0 (0)	26 (5.1)	508 (100.0)
Group 4 (12-18m)	16.0 (14.3)	33.8 (63.8)	20.8 (25.5)	8.1 (11.8)	488 (87.9)	31 (5.6)	1 (0.2)	35 (6.3)	555 (100.0)
Group 5 (18-24m)	9.8 (10.8)	41.1 (87.8)	26.4 (56.1)	7.1 (6.2)	262 (88.8)	19 (6.4)	1 (0.3)	13 (4.4)	295 (100.0)
Group 6 (24-30m)	8.8 (8.5)	35.4 (83.1)	25.5 (61.5)	7.7 (5.2)	127 (94.1)	4 (3.0)	0 (0)	4 (3.0)	135 (100.0)
Group 7 (30-36m)	8.5 (7.3)	18.3 (14.6)	18.3 (14.6)	11.8 (10.5)	238 (92.6)	19 (10.7)	0 (0)	0 (0.0)	257 (100.0)
Group 8 (36-42m)	4.9 (5.0)	21.6 (19.3)	21.6 (19.3)	10.5 (7.5)	181 (90.5)	19 (9.5)	0 (0)	0 (0.0)	200 (100.0)
All CYP	15.5 (14.4)	28.7 (60.3)	19.3 (29.8)	7.2 (9.3)	4402 (91.7)	214 (4.5)	9 (0.2)	177 (3.7)	4802 (100.0)

Table S2. Baseline mixed regression models examining association of admission group i.e. time between Arena event and joining Resilience Hub on total IES, DEP and CYP-GAD score during follow-up

Admission Group (Time to register with Hub post Arena event)	IES			DEP			CYP-GAD		
	Baseline effect	Baseline effect plus interaction		Baseline effect	Baseline effect plus interaction		Baseline effect	Baseline effect plus interaction	
	Coef (95% CI)	Coef (95% CI)		Coef (95% CI)	Coef (95% CI)		Coef (95% CI)	Coef (95% CI)	
All	-0.11 (-0.17, -0.05)	-0.09 (-0.17, -0.01)		0.07 (0.03, 0.10)	0.10 (0.05, 0.14)		-0.01 (-0.04, 0.02)	0.01 (-0.03, 0.04)	
	Effect	Effect	Interaction	Effect	Effect	Interaction	Effect	Effect	Interaction
Group 2 (6-9m)	2.91 (0.49, 5.33)	3.66 (0.73, 6.61)	-0.06 (80.20, 0.08)	0.77 (-0.61, 2.15)	1.94 (0.27, 3.61)	-0.10 (-0.18, -0.02)	1.07 (0.14, 2.01)	1.64 (0.45, 2.84)	-0.05 (-0.11, 0.01)
Group 3 (9-12m)	0.86 (-2.48, 4.20)	0.43 (-4.89, 5.76)	0.03 (-0.25, 0.30)	0.28 (-1.58, 2.15)	1.80 (-1.24, 4.84)	-0.10 (-0.26, -0.05)	0.98 (-0.33, 2.29)	1.71 (-0.51, 3.93)	-0.05 (-0.17, 0.07)
Group 4 (12-18m)	0.12 (-3.33, 3.57)	1.28 (-3.81, 6.38)	-0.09 (-0.37, 0.20)	1.05 (-0.91, 3.01)	0.37 (-2.53, 3.26)	0.05 (-0.11, 0.21)	1.51 (0.17, 2.84)	1.58 (-0.05, 3.67)	-0.01 (-0.13, 0.11)
Group 5 (18-24m)	1.64 (-2.98, 6.25)	-0.52 (-7.14. 6.10)	0.25 (-0.29, 0.79)	1.70 (-0.84, 4.23)	1.87 (-1.80, 5.55)	-0.01 (-0.31, 0.29)	1.17 (-0.62, 2.95)	1.51 (-1.29, 4.31)	-0.03 (-0.26, 0.20)
Group 6 (24-30m)	-0.57 (-5.90, 4.75)	-0.65 (-9.96, 8.66)	0.02 (-0.99, 1.04)	1.23 (-1.69, 4.15)	0.84 (-4.25, 5.93)	0.07 (-0.47, 0.62)	1.40 (-0.61, 3.41)	-0.01 (-3.69, 3.68)	0.20 (-0.21, 0.60)
Group 7 (30-36m)	-0.84 (-9.30, 7.62)	-8.13 (-20.12, 3.86)	1.53 (-0.23, 3.29)	3.69 (-1.12, 8.49)	-0.23 (-7.60, 7.14)	0.74 (-0.27, 1.75)	2.05 (-1.32, 5.43)	0.96 (-4.40, 6.32)	0.21 (-0.53, 0.94)
Cons	3.01(2.44,3.95)		10.19 (7.15, 13.23)	2.55 (1.56, 3.55)		2.24 (1.20, 3.28)	3.22 (2.34, 4.09)		3.04 (2.13, 3.95)
ICC*	0.474	0.474		0.453	0.444		0.359	0.357	
Global p-value**		0.534			0.136			0.664	

Table S3. Baseline mixed regression models examining association of admission group i.e. time between Arena event and joining Resilience Hub on total PG-GAD and SEP score during follow-up

Admission Group (Time to register with Hub post Arena event)	PG-GAD			SEP		
	Baseline effect	Baseline effect plus interaction		Baseline effect	Baseline effect plus interaction	
	Coef (95% CI)	Coef (95% CI)		Coef (95% CI)	Coef (95% CI)	
All	0.01 (-0.01, 0.04)	0.34 (0.003, 0.07)		-0.001 (-0.02, 0.02)	0.004 (-0.02, 0.03)	
	Effect	Effect	Interaction	Effect	Effect	Interaction
Group 2 (6-9m)	0.51 (-0.33, 1.35)	1.28 (0.24, 2.31)	-0.07 (-0.12, -0.01)	0.38 (-0.18, 0.94)	0.84 (0.11, 1.58)	-0.04(-0.08,0.001)
Group 3 (9-12m)	0.57 (-0.58, 1.71)	0.83 (-1.03, 2.69)	-0.02 (-0.12, 0.74)	0.28 (-0.51, 1.07)	-0.54 (-1.90, 0.81)	0.05 (-0.02, 0.13)
Group 4 (12-18m)	1.83 (0.69, 2.97)	2.06 (0.23, 3.89)	-0.02 (-0.13, 0.84)	0.85 (0.10, 1.60)	0.55 (-0.75, 1.85)	0.02 (-0.06, 0.10)
Group 5 (18-24m)	0.25 (-0.05, 1.66)	-0.37 (-2.54, 1.79)	0.07 (-0.10, 0.28)	1.28 (0.28, 2.27)	0.60 (-1.02, 2.21)	0.07 (-0.06, 0.21)
Group 6 (24-30m)	1.92 (0.09, 3.74)	2.40 (-1.01, 5.82)	-0.05 (-0.38, 0.28)	0.62 (-0.80, 2.04)	2.76 (-0.72, 6.24)	-0.23 (-0.59, 0.12)
Group 7 (30-36m)	0.96 (-2.19, 4.12)	1.69 (-2.81, 6.19)	-0.12 (-0.75, 0.51)	-1.81 (-4.30, 0.68)	-0.31 (-3.65, 3.03)	-0.31 (-0.78, 0.16)
Cons	2.37 (1.64, 3.09)		2.12 (1.36, 2.88)	5.55 (4.69, 6.41)		5.48 (4.60, 6.35)
ICC*	0.401	0.404		0.242	0.235	
Global p-value**		0.301			0.069	
